# Metabolic Regulation by the Hypothalamic Neuropeptide, Gonadotropin-Inhibitory Hormone at Both the Central and Peripheral Levels

**DOI:** 10.3390/cells14040267

**Published:** 2025-02-12

**Authors:** You Lee Son, Simone L. Meddle, Yasuko Tobari

**Affiliations:** 1 Division of Stem Cell Biology, Institute for Genetic Medicine, Hokkaido University, Kita 15, Nishi 7, Kita-ku, Sapporo 060-0815, Japan; 2 The Roslin Institute and Royal (Dick) School of Veterinary Studies R(D)SVS, University of Edinburgh, Easter Bush Campus, Midlothian EH25 9RG, UK; simone.meddle@roslin.ed.ac.uk; 3 Center for Human and Animal Symbiosis Science, Department of Animal Science and Biotechnology, School of Veterinary Medicine, Azabu University, Fuchinobe 1-17-71, Chuo-ku, Sagamihara 252-5201, Japan; tobari@azabu-u.ac.jp

**Keywords:** gonadotropin-inhibitory hormone (GnIH), RFamide-related peptide-3 (RFRP-3), metabolism, food intake, hypothalamus, adipose tissue

## Abstract

Gonadotropin-inhibitory hormone (GnIH) is well-established as a negative regulator of reproductive physiology and behavior across vertebrates, acting on the hypothalamic–pituitary–gonadal (HPG) axis; however, recent data have also demonstrated its involvement in the control of metabolic processes. GnIH neurons and fibers have been identified in hypothalamic regions associated with feeding behavior and energy homeostasis, with GnIH receptors being expressed throughout the hypothalamus. GnIH does not act alone in the hypothalamus, but rather interacts with the melanocortin system, as well as with other neuropeptides. GnIH and its receptors are also expressed in peripheral tissues involved in important metabolic functions. Therefore, the local action of GnIH in peripheral organs, including the pancreas, gastrointestinal tract, gonad, and adipose tissue, is also suggested. This review aims to provide a comprehensive summary of the emerging role of GnIH in metabolic regulation at both the central and peripheral levels.

## 1. Introduction

Since the gonadotropin-inhibitory hormone (GnIH) was first discovered in 2000 as the hypothalamic neuropeptide that actively inhibits gonadotropin release [1], much evidence now supports GnIH as having a role as a key neurohormone to inhibit reproduction by regulating the hypothalamic–pituitary function. Given that a complex reciprocal mechanism exits between the reproductive axis and other endocrine systems, accumulating evidence has also suggested the involvement of GnIH in the adrenal and thyroid axes. Moreover, recent evidence suggests that GnIH also influences feeding behavior and energy homeostasis. GnIH-mediated metabolic control may occur at multiple levels. Although most interactions between metabolic factors and GnIH occur in the hypothalamus, the direct influence of GnIH on peripheral metabolic tissues has recently been recognized. In this review, we aim to highlight the endocrine role of central and peripheral GnIH in the regulation of metabolic processes.

## 2. Overview of GnIH and Its Impact on the Endocrine System

### 2.1. GnIH and Its Receptors

GnIH, the peptide having a C-terminal RFamide motif, Ser-Ile-Lys-Pro-Ser-Ala-Tyr-Leu-Pro-Leu-Arg-Phe-NH_2_ (SIKPSAYLPLRFamide), was initially isolated from the Japanese quail (*Coturnix japonica*) hypothalamus, and it was shown that GnIH inhibited gonadotropin release from the Japanese quail cultured anterior pituitary gland [1]. The GnIH precursor gene encodes one GnIH and two GnIH-related peptides (GnIH-RP-1 and GnIH-RP-2), which possess a common C-terminal LPXRFamide (X = L or Q) motif in all avian species studied [2,3,4,5,6]. Subsequently, GnIH peptides have been identified in all vertebrate classes that share an LPXRFamide (X = L or Q) motif at their C-termini [7,8,9], like avian GnIH and GnIH-RPs, and are thus also known as RFamide-related peptides (RFRPs). The mammalian GnIH precursor gene is translated and cleaved into at least two GnIH peptides, RFRP-1 (also known as neuropeptide SF, NPSF) and RFRP-3 (also known as neuropeptide VF, NPVF) [7,8,9]; RFRP-2 is not an LPXRFamide peptide.

Two G protein-coupled receptors, GPR147 (also known as neuropeptide-FF receptor 1, NPFFR1; NPFF1; NPFF1R1; OT7T022) and GPR74 (also known as NPFFR2; NPFF2; NPGPR; HLWAR77) have been identified as GnIH receptors (GnIH-Rs) [3,10,11,12]. Given the higher GnIH binding affinity for GPR147 than GPR74, it is postulated that GPR147 is the principal receptor for GnIH [3,11]. Both GnIH-Rs couple to Gα_i_, which inhibits the activity of adenylate cyclase (AC), thus reducing intracellular cAMP levels and protein kinase A (PKA) activity [10,13,14,15]. The molecular mechanism of GnIH-mediated cAMP inhibition was investigated using cellular model systems derived from the hypothalamus, anterior pituitary gland, and gonadal tissue [16,17,18]. The results indicate that inhibition by GnIH is through specific targeting of the AC/cAMP/PKA pathway.

### 2.2. Regulation of the Endocrine System by GnIH

GnIH is localized in various regions of the brain in many species, particularly in the diencephalon and mesencephalon, with a specific focus on the hypothalamic region [19,20,21,22]. GnIH neuronal cell bodies are found in the paraventricular nucleus (PVN) of birds [1,23,24]; in the dorsomedial hypothalamic area (DMH) of mice [25], hamsters [20,25,26], and rats [10,25,26,27]; in both the PVN and DMH of sheep [28]; in the dorsomedial nucleus (DMN), intermediate periventricular nucleus (IPe), PVN, and arcuate nucleus (ARC) of pigs [29]; and in the IPe of macaques [30]. GnIH neuronal projections are also identified throughout the brain, including the preoptic area (POA), lateral septum, arcuate nucleus (ARC), and anterior hypothalamus in mammals [22,25,31,32]. Similarly, GnIH-Rs are also expressed throughout the hypothalamus, specifically in the POA, rostral periventricular area of the third ventricle, and ARC. The widespread localization of GnIH neurons and GnIH-Rs within the hypothalamic region strongly suggests multiple neuroendocrine functions of the GnIH system.

#### 2.2.1. Role of GnIH on the Reproductive Axis

The control of reproduction depends on the intact HPG axis. Reproductive neuropeptides synthesized in hypothalamic neurons, including the well-known gonadotropin-releasing hormone (GnRH), kisspeptin, and GnIH, regulate the release of pituitary gonadotropin, follicle stimulating hormone (FSH), and luteinizing hormone (LH) that, in turn, control gonadal functions and the production of sex hormones. Extensive studies have established that GnIH peptides reduce the synthesis and/or release of gonadotropin (reviewed in refs. [7,8,33,34,35,36]). At the hypothalamic level, GnIH inhibits the activity of GnRH and/or kisspeptin neurons. Based on the morphological evidence, GnIH has been considered to act at the most upstream level of the HPG axis, as described below.

GnRH stimulates the release of gonadotropin and plays a fundamental role in controlling physiological aspects of the reproductive process. The projection of GnIH neurons to GnRH neurons is the most conserved characteristic of GnIH neurons across species. GnIH neuronal axon terminals form both axo-somatic and axo-dendritic contacts with GnRH neurons, which express GnIH-Rs in the POA [20,25,37,38,39,40]. Direct application of GnIH to hypothalamic brain slices decreased the firing rate of a subpopulation of GnRH neurons [41]. Electrophysiological recordings also support this observation that a direct postsynaptic inhibition of GnRH neuronal firing may occur via GnIH [42]. Additionally, intracerebroventricular (ICV) administration of GnIH suppressed cFOS activity in GnRH neurons, indicating the direct suppressive effects of GnIH on the regulation of GnRH neurons.

Kisspeptin serves as a stimulatory regulator for GnRH release across various vertebrate species, excluding birds. In mammals, kisspeptin neurons are found in the anteroventral periventricular nucleus (AVPV) and ARC. AVPV kisspeptin neurons are regarded as a target of estrogen-positive feedback to induce a preovulatory GnRH/LH surge, whereas ARC kisspeptin neurons are involved in the negative regulation of GnRH/LH secretion by sex steroids. In mice, only a small portion (5–15%) of kisspeptin neurons in the AVPV expresses GPR147 or GPR74, whereas a higher co-expression with either of the GnIH-Rs is observed in the ARC kisspeptin neurons. Approximately 35% of kisspeptin neurons in the ARC are contacted by GnIH neuronal fibers [43], suggesting that GnIH may directly modulate the kisspeptin neuronal activity associated with the generation of pulsatile GnRH/LH secretions.

GnIH neuronal fibers are also observed in the median eminence (ME) and control anterior pituitary function via GnIH-Rs expressed in the gonadotropes [1,19,23,28,30,40,44]. Although there is some debate regarding whether GnIH can act directly on the anterior pituitary gland in some species, evidence from numerous studies indicates that GnIH can decrease pituitary gonadotropin synthesis and/or release in many species [22,28,38,45,46,47,48].

While the inhibitory effect of GnIH on reproduction is mainly accomplished at the hypothalamic–pituitary levels, GnIH and GnIH-Rs are also expressed in steroidogenic cells and germ cells in the gonads, testis, and ovary, indicating that autocrine or paracrine mechanisms of the GnIH system exist to control gonadal functions by inhibiting sex steroid production and gametogenesis [16,49,50,51]. Therefore, GnIH is a key regulator that inhibits each level of the HPG axis by controlling the activities of hypothalamic GnRH and kisspeptin neurons, pituitary gonadotropin secretion, and gonadal function. It should be noted that although GnIH exerts an inhibitory effect on the HPG axis at the cellular level, GnIH or GnIH-R (GPR147) knockout (KO) mice do not exhibit abnormalities in their reproductive phenotypes under normal conditions. Pubertal timing and fertility were unaffected in GnIH KO mice [52] (Tsutsui and colleagues, unpublished observation) and in GPR147 KO mice [53]. The role of GnIH as a negative regulator in the reproductive system suggests that, under healthy, normal conditions, GnIH does not actively inhibit reproductive processes; however, if GnIH levels are elevated under abnormal endocrine conditions, the absence of the GnIH system in these KO models could potentially reverse the effects of such imbalances. This will be further discussed in the following section.

#### 2.2.2. Involvement of GnIH in the Stress Axis

The stress system, known as the hypothalamic–pituitary–adrenal (HPA) axis, is closely linked to the HPG axis. Prolonged exposure to stress can disrupt the equilibrium between the HPA and HPG axes, resulting in reduced reproductive function and disorders associated with infertility. Stress response is characterized by the hypothalamic release of corticotropin-releasing hormone (CRH) and arginine vasopressin (AVP), which, in turn, induce the release of adrenocorticotropic hormone (ACTH) from the anterior pituitary. ACTH causes the adrenal cortex to secrete glucocorticoids (GC), such as corticosterone in birds and rodents. When GC levels reach a certain concentration, GC exerts negative feedback via CRH, AVP, and ACTH to end the stress response, thereby returning to the systemic homeostasis. GnIH neurons in the PVN directly contact with CRH [54]; furthermore, the CRH receptor-1 is expressed in GnIH neurons [55], and GnIH neurons express the GC receptor (GR) [55,56], indicating that adrenal GC can mediate the stress effect through direct action on GnIH neurons.

Both the HPA and HPG axes exhibit striking sex differences, and there are controversial effects depending on the species and stress paradigm. Nonetheless, most experimental stress protocols, such as restraint/immobilization [55,57,58,59,60,61,62], nutritional/metabolic [53,63,64,65], and thermal stress [66,67], have been shown to activate GnIH and/or GnIH-Rs in the hypothalamus and/or gonad, which are associated with negatively related gonadotropin or sex hormone levels (reviewed in ref. [68]). Importantly, the suppressive effects of stress on HPG activity are prevented by the ablation of GnIH neurons [61], as well as the knockdown of GnIH [57], GPR147 KO [53], or administration of the GPR147 antagonist [64]. These findings suggest that GnIH plays a crucial role in mediating stress-induced reproductive dysfunction; in addition, the pharmacologically-induced stress status, induced by administration of ACTH or corticosterone, increases the expression of GnIH or GnIH-Rs [56,69,70]. Importantly, GR is recruited to the GnIH promoter region in response to corticosterone [55,56], further supporting the direct activation of GnIH in response to stress.

GnIH also exerts a stimulatory effect on the HPA axis. Furthermore, ICV administration of GnIH significantly elevated serum corticosterone levels in conscious male rats [71]. Mice that received chronic infusions of GnIH exhibited anxiogenic effects and demonstrated elevated basal-circulating corticosterone levels [72]. It is noteworthy that a highly selective GPR147 antagonist, GJ14, effectively impeded GnIH-induced corticosterone release and CRH neuronal activation, while also reversing the anxiogenic impact of GnIH [72]. Furthermore, acute stimulation of GnIH neurons resulted in a significant, dose-dependent release of corticosterone [61]. These results indicate the existence of a positive feedback loop, whereby stressful stimuli activate the GnIH system, which, in turn, further activates the HPA axis. The expression of GPR147 in the PVN, where the CRH/AVP neurons are located, may provide support for the potential modulatory action of GnIH in this positive feedback loop.

#### 2.2.3. Involvement of GnIH in the Thyroid Axis

Thyroid hormones (THs; thyroxine, T_4_, and triiodothyronine, T_3_) are well-known as regulators of metabolism, development, and growth. They play a vital role in the normal development and function of the reproductive system, suggesting close interactions between the hypothalamic–pituitary–thyroid (HPT) and HPG axes. Therefore, thyroid disorders, such as hypothyroidism and hyperthyroidism, cause abnormal reproductive function. As elevated TH levels are known to activate GnRH neurons and indirectly suppress the activity of GnIH [73,74,75], T_4_-induced hyperthyroidism leads to a decrease in hypothalamic GnIH expression, and T_3_ treatment suppresses GnIH mRNA expression in hypothalamic explants [52]. In contrast, hypothyroidism induced by long-term administration of propylthiouracil (PTU), which inhibits the production of new TH, in juvenile female mice results in delayed pubertal onset, with increased hypothalamic GnIH expression and decreased pituitary–gonadal activity; the effect of hypothyroidism in delaying pubertal onset is prevented by GnIH KO [52]. Although, to date, limited information is available for the TH-mediated GnIH regulation, the expression of TH receptors in GnIH neurons [52] suggests a direct involvement of GnIH in mediating HPT–HPG interactions.

## 3. Metabolic Regulation by Hypothalamic GnIH

The hypothalamus is the primary target of metabolic programming and the principal regulatory center of energy metabolism, acting through both neuronal and hormonal mechanisms, and each hypothalamic area has a distinct role in the metabolic regulation [76,77]. The wide distribution of GnIH fibers and GnIH-Rs throughout regions of the hypothalamus involved in appetite and energy homeostasis, including the ARC, PVN, lateral hypothalamus, and ventromedial nucleus (as described in Section 2.2), supports the significance of GnIH beyond the reproductive axis. Therefore, there have been attempts to determine whether GnIH administration would affect metabolism.

### 3.1. Effect of Central GnIH Administration on Feeding Behavior

It has been confirmed that central GnIH administration affects the appetite in many species. The stimulatory effect of GnIH on food intake was first demonstrated in domestic chicken chicks [78]. Tachibana et al. showed that ICV injection of GnIH stimulates food intake, whereas anti-GnIH antiserum injection significantly inhibits food intake in food deprivation-induced feeding [78]. Subsequent studies have demonstrated that GnIH exerts stimulatory effects on food intake in other bird species and mammals including mice, hamsters, rats, sheep, and cynomolgus monkeys, as evidenced by an increase in food intake following ICV injection of GnIH [22,79,80,81,82,83,84]; however, other factors also play a role, such as sex or duration of GnIH administration. For example, chronic injections of GnIH did not stimulate food intake in female hamsters [83], and this was also observed with acute injections in male hamsters [84] and rats [85].

A single study has reported the inhibitory effect of GnIH (specifically RFRP-1) on feeding behavior through direct brain microinjection of GnIH into the amygdala [86]. The amygdala plays an important role in the regulation of food intake and body weight; GnIH-immunoreactive fibers and GPR147 are found in the amygdala [87]. Kovács et al. showed that intra-amygdaloid microinjection of GnIH decreased liquid food intake in rats, and this effect was prevented by pretreatment with the GPR147 antagonist RF9 in the amygdala [86]. The metabolic effects of central GnIH administration described above are summarized in Table 1.

### 3.2. Interaction with the Melanocortin System

The hypothalamus achieves energy homeostasis mainly through the melanocortin system, which is located in the ARC and DMH. The melanocortin system has direct connections to the PVN and other brain regions to regulate feeding behavior as well as energy homeostasis. The melanocortin system involves the orexigenic agouti-related peptide (AgRP)/neuropeptide Y (NPY) neurons, and the anorexigenic proopiomelanocortin (POMC)/cocaine-and-amphetamine-regulated transcript (CART) neurons. GnIH fibers form close apposition with the NPY and POMC neurons in the ARC [30,54,88]. Although GnIH is considered to have a stimulatory effect on feeding behavior, the direct role of GnIH on the melanocortin system has yielded conflicting results.

Consistent with the orexigenic function of GnIH, GnIH has been found to inhibit the firing rate of POMC neurons in hypothalamic slices from mice [88,89]. Similarly, hypothalamic POMC expression was shown to be increased in GPR147 KO male mice, which exhibited a decline in spontaneous food intake, further suggesting the orexigenic action of endogenous GnIH/GnIH-R signaling [90]. The orexigenic effect was also observed in male mice [82] and hamsters [83] after chronic GnIH ICV injection; however, the expression of hypothalamic genes involved in metabolism, such as POMC, NPY, or AgRP, was not altered.

Despite its well-documented ability to stimulate food intake in various species, including mice, GnIH showed a predominantly inhibitory effect on the electrophysiological and functional activity of NPY neurons (80% of those tested) in mice [88]. Furthermore, GnIH also inhibited the secretion of NPY in incubated hypothalamic slices [88]; however, GnIH excited less than 20% of NPY neurons, and a similar percentage of NPY neurons received close input from GnIH-immunoreactive fibers. These findings suggest that the control of NPY neurons in the mouse ARC is complex.

### 3.3. Role of GnIH in Mediating the Effects of Leptin and Ghreilin on Food Intake

The adipose-derived hormone leptin has strong effects on the hypothalamic regulation of satiety, energy expenditure, and body weight. Leptin acts centrally through its specific receptor, LepR. Earlier studies have shown that leptin has little or no effect on GnIH neurons. Poling et al. showed that LepR mRNA is found to be co-expressed in ∼15% of GnIH neurons [91], but other research concluded that LepR mRNA is undetectable in semi-purified GnIH neuronal preparations [92]. Regardless of whether LepR is expressed in GnIH neurons or not, both groups indicated that leptin-deficient mice exhibit either a minor reduction in GnIH mRNA levels or no detectable difference compared to wild-type mice. Furthermore, the postnatal development of GnIH neurons appears to be unaffected by leptin deficiency [91]. Together, these results indicate that GnIH is unlikely to be an important neuronal pathway for the direct regulation of metabolism by leptin.

Ghrelin, which is secreted from the gastrointestinal (GI) tract, exerts orexigenic effects by acting on the hypothalamic ARC. Although ghrelin is primarily linked to the GI tract, some studies have reported ghrelin-immunoreactive cells in the hypothalamus [93,94], and the existence of ghrelin-producing neurons was reported in the ARC [95]. To date, the direct interaction between GnIH and ghrelin has not been extensively studied, and the potential impact of ghrelin on the activity of GnIH neurons is not well-understood. However, given the widespread distribution of GnIH and GnIH-Rs in the brain, it is hypothesized that GnIH neurons may project to ghrelin neurons in the ARC and contribute to the regulation of its central effects (reviewed in ref. [96]).

Despite the absence of direct evidence for their interactions in the hypothalamus, GPR147 KO male mice exhibited an altered food intake response induced by ICV administration of leptin and ghrelin [87]. Anorectic responses to leptin were exaggerated in GPR147 KO mice compared to wild-type mice during low-fat diet conditions. Conversely, orexigenic responses to ghrelin were blunted in GPR147 KO mice during high-fat diet (HFD) conditions. Thus, this study suggests the possible involvement of the GnIH system in the feeding response by these hormones, in which the effects of leptin and ghrelin might partially depend on the integrity of the GnIH/GPR147 signaling pathway.

## 4. Metabolic Regulation by Peripheral GnIH

Although GnIH is primarily produced in the hypothalamus, similar to other neuropeptides, it can potentially impact peripheral tissues expressing GnIH-Rs through endocrine signaling via the bloodstream, paracrine signaling for local regulation, or autocrine signaling for self-regulation. Recent evidence suggests a broader role for GnIH in peripheral tissues, as indicated by the expression of GnIH and/or GnIH-Rs in peripheral endocrine organs, not just the brain. Among these organs, the intra-gonadal role of GnIH has been well-investigated in relation to the integration of reproduction and metabolism (reviewed in ref. [97]). Thus, in this review, we focus on the potential role of GnIH in metabolic tissues, such as adipose tissue, pancreas, GI tract, and liver, where expression of the GnIH system is confirmed (Table 2). As this is a relatively new field of GnIH research, in this section we discuss the latest progress in peripheral metabolic control by the GnIH system.

### 4.1. Effect of Peripheral GnIH Administration

The peripheral administration of GnIH has been demonstrated to induce orexigenic effects that resemble those observed following central ICV injection. Chronic intraperitoneal (IP) injection of GnIH has been shown to increase food intake in male mice [104,105], as well as in both male and female rats [99], and female piglets [106]. These chronic IP injection models have shown the hyperphagia-induced weight gain, adiposity, and impaired glucose homeostasis, suggesting the potential involvement of GnIH in obesity-induced metabolic disorders and related reproductive dysfunction. However, it remains unclear whether peripheral GnIH can enter the brain via its receptor-mediated transport crossing the blood–brain barrier. Alternatively, IP-injected GnIH may act on the peripheral organs expressing GnIH-Rs. We have summarized the effect of IP injection of GnIH on metabolism in Table 3, and the changes in metabolic tissues induced by GnIH injection are described in the following section.

### 4.2. Adipose Tissue

**Figure 1:**
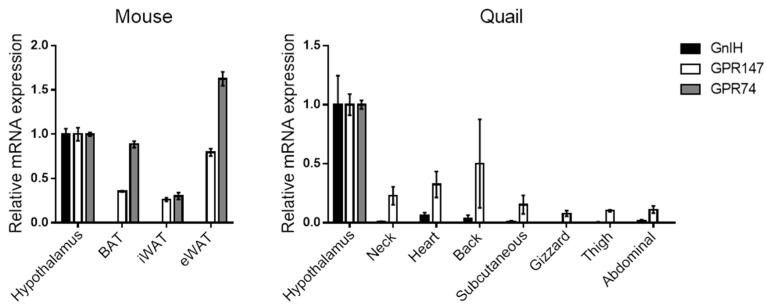
Expression of GnIH and GnIH-Rs in various adipose tissues isolated from mouse (**left**) and Japanese quail (**right**). Mouse adipose tissues include interscapular BAT, subcutaneous iWAT, and visceral eWAT. Japanese quail adipose tissues are collected close to the indicated depot and organ. RT-PCR was performed according to our previous report [17]. Expression levels are shown by setting “Hypothalamus” to 1.

It appears that GnIH itself is not produced in adipose tissues, and we have also confirmed that GnIH mRNA is not detected in various adipose depots in both mice and Japanese quail (Figure 1). However, GnIH-Rs have been observed in human [98] and rat [99] adipose tissues, suggesting the direct effect of GnIH on adipose tissues via its receptors. Our expression profiling also indicates that GPR147 and GPR74 are abundantly expressed in mouse brown and white adipose tissues (BAT and WATs), with higher levels of GPR74 than GPR147 (Figure 1, left). In Japanese quail adipose tissues, GPR147 is predominantly expressed in all depots tested, compared to GPR74 (Figure 1, right). In human samples, both GPR147 and GPR74 were expressed at the protein level in freshly isolated mature adipocytes, as well as in in vitro-differentiated adipocytes from the omental and subcutaneous region [98]. GPR74 mRNA expression levels were particularly higher in adipose tissue from obese subjects, compared to non-obese subjects [98]. In mouse 3T3-L1-differentiated adipocytes, both GnIH-Rs were also expressed [100]. Given the characteristics of GnIH-Rs, which are coupled with Gα_i_ to inhibit the cAMP pathway, it can be hypothesized that a higher gene expression of either receptors may be associated with lower lipolysis activity by inhibiting the noradrenaline/cAMP/PKA pathway [108]. Indeed, in in vitro-differentiated adipocytes isolated from human subjects, RFRP-1 was indicated to be important for the regulation of lipolysis, probably acting through GPR74 and GPR147 [98]. Although the direct effect of RFRP-3 was not investigated in this study, its binding affinity to these receptors suggests that RFRP-3 may have a similar effect to RFRP-1 on lipolysis.

Chronic GnIH treatment causes an increase in the mass of adipose tissue [82,99,104,105,106] in many species. Chronic ICV infusion of GnIH for 13 days increased BAT mass, resulting in a WAT-like appearance. BAT is the thermoregulatory organ to enhance energy expenditure; thus, the observed decreases in energy expenditure and core body temperature could be explained by the impaired BAT function in GnIH-injected mice [82]. Although centrally injected GnIH did not change WAT mass, peripheral administration of GnIH significantly increased WAT mass and altered its function related to glucose and lipid metabolism. Anjum et al. showed that in vivo (IP injection for 8 days) and in vitro treatment of GnIH resulted in upregulation of glucose transporter 4 (GLUT4), a major insulin-stimulated glucose transporter, and increased triglyceride uptake in abdominal visceral WAT, contributing to obesity-related metabolic abnormalities in male mice [104]. Similarly, chronic IP injection of GnIH for 14 days in male mice led to hyperlipidemia, hyperglycemia, glucose intolerance, and insulin resistance through changes in the expression of glucose and lipid metabolism-related genes in perigonadal visceral WAT [105]. A similar set of effects was observed in rats [99] and female piglets [106] after IP administration of GnIH, including an increase in WAT mass and impaired glucose transport and insulin signaling in WAT. These effects were associated with a disruption in whole-body glucose homeostasis. The molecular mechanism of GnIH-induced impaired glucose metabolism via inhibition of AKT-GSK3-β signaling is suggested [99]; however, it remains unclear whether this effect is directly mediated by GnIH-Rs in WAT. Further studies using GnIH-Rs antagonists or the overexpression/knockdown of GnIH-Rs are required to further elucidate the direct involvement of GnIH via its receptors.

### 4.3. Pancreas

Given the effect of GnIH on food intake and glucose homeostasis, it has been hypothesized that GnIH/GnIH-Rs may play a role in the pancreas, a vital organ in regulating blood glucose levels through the secretion of insulin and glucagon. In addition, other RFRP members, 43RFa and 26RFa, have been shown to promote the survival of INS-1E rat pancreatic β-cells and human pancreatic islets [109], suggesting the possible involvement of GnIH in the pancreatic regulation. Indeed, abundant GnIH-immunoreactive cells were found to be concentrated in the pancreatic islets of rats [99] and male piglets [29]. In addition, GPR147 expression was also confirmed in the islets of rats [99] and mice [101]. Specifically, intense GnIH-immunoreactivity was observed in some α-cells, but not in β-cells, indicating that GnIH colocalized primarily with glucagon [99]. In contrast, moderate GPR147 immunoreactivity was observed around the islet and endocrine β-cells of the pancreas and weak immunoreactivity with α-cells, indicating that GPR147 colocalized primarily with insulin in the pancreatic islets [99].

Using αTC1, a mouse islet α-cell line which was also validated to express GPR147, it was reported that GnIH promoted the survival of α-cells under conditions of hyperglycemia and serum starvation [101]. Mechanistically, GnIH activated PI3K/AKT and ERK1/2 signaling cascades, and treatment with RF9 (GPR147 antagonist) blocked the activation of both pathways, thereby indicating the possible role of GnIH in promoting α-cell survival, probably via GPR147. In vivo studies have shown that IP-injected GnIH induces changes in islet histomorphology, thereby altering insulin and glucagon secretion from the pancreas, which contributes to hyperglycemia and insulin resistance in GnIH-treated models. In rat models, chronic GnIH treatment for 14 days resulted in a dramatic induction of pancreatic islet hyperplasia and significantly inhibited insulin secretion, accompanied by elevated glucagon secretion [99]. Similarly, female piglets treated with GnIH for 14 days showed increased pancreatic mass and islet hypertrophy, along with hyperinsulinism and hyperglucagon [106]. Taken together, these in vitro and in vivo findings suggest a direct action of GnIH on pancreatic islets, potentially via GPR147, to regulate glucose homeostasis.

### 4.4. GI Tract

Despite the limitations of a few studies, GnIH-immunoreactive cells were found to be widely distributed throughout the GI tract, including the esophagus, stomach, small intestine, and large intestine, in male piglets [29]. GnIH immunoreactivity was found in the mucosal layers and the tunica muscularis throughout the digestive tract, which may suggest its presence in epithelial cells, smooth muscle cells, or potentially in neurons and glial cells within the enteric nervous system (ENS), although the exact cell types were not specified in this study [29]. Additionally, low levels of GPR147 mRNA were detected in female pigs [102]. Both GnIH and GPR147 mRNA were also observed at low levels in the stomach, ileum, and colon of mice [103]. Xu et al. recently reported the potential role of GnIH in stress-induced intestinal dysfunction in domestic chickens [107]. In this study, chronic IP injection of GnIH for 14 days not only induced intestinal and systemic stress, but also led to the disruption of the physical, chemical, and microbial barriers of the intestine, as well as an increase in intestinal inflammation. The in vivo findings were further validated by treatment with GnIH in jejunal explants in vitro, revealing that GnIH directly damaged the intestinal barriers.

### 4.5. Liver and Skeletal Muscle

GnIH was not detected in the liver and muscle of rats [99] and male piglets [29], whereas GPR147 was expressed in the liver and skeletal muscle of rats at both mRNA and protein levels, to a similar extent as in WAT [99]. Hou et al. conducted a comparative analysis of GnIH-mediated effects on the liver, skeletal muscle, and WAT [99]. Chronic GnIH injection (IP for 14 days) resulted in similar effects in the liver and WAT, including decreased insulin receptor and GLUT4 mRNA expression and increased proinflammatory gene expression. Importantly, these changes were more evident in the liver than in WAT, implying that the liver might also serve as the target for GnIH action. Altered signaling pathways following GnIH administration showed similar patterns, marked by increased AKT phosphorylation and inhibited GSK3-β phosphorylation in both the liver and WAT. In contrast, skeletal muscle exhibited a minimal response in GLUT4 and proinflammatory gene expression, along with inhibited AKT phosphorylation after GnIH administration. The changes observed in each tissue following GnIH administration are believed to contribute to the increased systemic insulin resistance and inflammatory response.

## 5. Nutritional Status and GnIH: Obesity and Fasting

In genetically obese (ob/ob) and HFD-induced obese mouse models, the expression of GnIH itself remained unchanged [92]. However, GPR147 KO female mice exhibited increased body weight gain during long-term HFD feeding compared to WT, associated with increased fat mass and decreased total energy expenditure [90]. In the same study, HFD-fed GPR147 KO male mice showed impaired glucose tolerance and insulin sensitivity without changes in body weight and body composition. These data suggest that GnIH/GPR147 signaling regulates metabolic homeostasis in a sexually dimorphic manner.

Increased expression of GnIH has been observed in response to fasting. In female hamsters, there was a gradual increase in the activation of GnIH neurons (cFOS activity) in the DMH, concomitant with an increase in the duration of food restriction for 12 days. These gradual changes in the activation of GnIH neurons were remarkably similar to the changes in appetitive ingestive behavior (food hoarding) and were the exact opposite of changes in appetitive sexual behavior (male preference) [65,110,111]. In male zebra finches, 10 h of fasting increased gonadal GnIH mRNA expression, but did not affect the hypothalamic GnIH expression at both immunoreactivity and mRNA levels [112]. It should be noted that fasting/food deprivation is a widely used stress protocol to induce the endocrine and metabolic changes in experimental animals, accompanied by elevated circulating corticosterone levels [113,114,115]. Thus, increased GnIH expression induced by the fasting condition may be attributable to the direct effect of corticosterone on the GnIH system, as described in Section 2.2.2.

## 6. Conclusions

**Figure 2:**
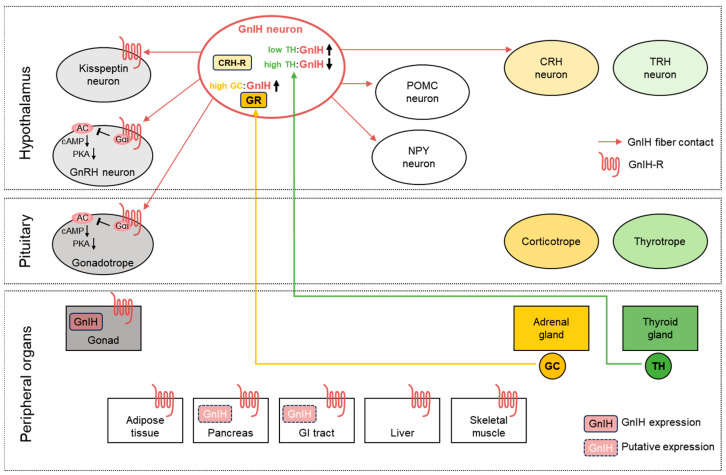
A schematic diagram summarizing the possible involvement of the GnIH system in endocrine axes and peripheral regulation. In the HPG axis (gray), GnIH neuronal fibers project to kisspeptin neurons and GnRH neurons in the hypothalamus, as well as to gonadotropes in the pituitary. The Gα_i_-coupled GnIH-Rs specifically inhibit the AC/cAMP/PKA signaling pathway, as demonstrated by in vitro GnRH neuron and gonadotrope models. In interactions with the HPA axis (yellow), stress-induced GC (high GC) can directly activate GnIH neurons via GR, leading to reproductive dysfunction. In interactions with the HPT axis (green), hypothalamic GnIH expression is increased by hypothyroidism (low TH), resulting in delayed puberty, whereas it is decreased by hyperthyroidism (high TH). In metabolic regulation by the GnIH system, GnIH has an orexinergic effect to increase food intake, possibly through regulating the activity of POMC and NPY neurons in the hypothalamus, as GnIH neuronal fibers make direct contact with these neurons. The GnIH system is also found in various peripheral tissues, such as adipose tissue, pancreas, GI tract, liver, and skeletal muscle, suggesting its involvement in the control of metabolic processes beyond reproduction. CRH, corticotropin-releasing hormone; CRH-R, corticotropin-releasing hormone receptor; GC, glucocorticoid; GR, glucocorticoid receptor; TH, thyroid hormone; POMC, proopiomelanocortin; NPY, neuropeptide Y.

Since its discovery in 2000, an increasing number of functions have been identified for GnIH neuropeptide. As summarized in Figure 2, the widespread expression of GnIH and its receptors, in both the brain and peripheral tissues, suggests that it plays an expanding range of roles in general physiology, beyond its originally reported role in reproduction. For example, the orexigenic function of GnIH is now well-accepted, as evidenced by the many studies showing increased food intake following ICV administration. However, the close contact of GnIH fibers with NPY and POMC neurons does not fully explain the orexigenic effect, since their neuronal activities or gene expression patterns were not associated with the orexigenic changes caused by GnIH treatment in vivo and in vitro. Furthermore, there is no evidence for GnIH-R expression in these neurons. Therefore, the detailed mechanism linking the central GnIH system and feeding behavior requires further investigation. As described above, chronic IP administration of GnIH has been found to induce metabolic abnormalities, accompanied by significant alteration in peripheral tissue metabolism. The increased food intake observed following IP GnIH injection may be the primary contributor to weight gain and adiposity, but it is unclear how IP-injected GnIH stimulates food intake and which organ is most responsible for these GnIH-induced metabolic disorders. Currently, there is insufficient evidence for the direct action of GnIH via its receptors, other than the fact that GnIH/GnIH-Rs are expressed in the peripheral metabolic tissues. Further cellular, animal, and human studies are required to fully elucidate GnIH’s effects across various physiological contexts. In parallel, the detailed molecular mechanism of the GnIH action should be investigated to explore its potential as a therapeutic target.

## Figures and Tables

**Table 1:** Effect of central GnIH administration on metabolism. Avian GnIH peptide or mammalian RFRP-3 peptide is used for ICV injection, except for intra-amygdaloid microinjection of RFRP-1 peptide.

Animal Models	Injection	Metabolic Effects	Ref.
Male domestic chicken chick	Single ICV (0.3, 0.9, or 2.6 nmol)	• Increased food intake at 1 and 2 h	[78]
	Single ICV of anti-GnIH antiserum	• Reduction in food deprivation-induced feeding • No effect on ad libitum feeding	
Male domestic chicken chick	Single ICV (0.9, 2.6, or 7.8 nmol)	• Increased food intake at 0.5 to 2 h • No effect on water intake • Increased feeding pecks at 5 min to 30 min	[81]
Adult male Pekin duck	Single ICV (100 ng)	• Increased food intake at 2 h	[80]
Adult male mouse	Single ICV (25, 50, or 100 ng)	• Increased food intake at 0.5, 1, 2 h	[79]
Adult male mouse	ICV (6 nmol/day) for 13 days	• Increased food intake, BW, BAT and liver mass • No effects on WATs and muscle mass • Decreased O_2_/CO_2_ metabolism, energy expenditure, and core body temperature ^1^ • No effects on locomotor activity	[82]
SD-adapted male hamster	ICV (8.25 pmol/h) for 5 weeks	• Increased BW and food intake • Increased circulating insulin and leptin • No effects on hypothalamic metabolic genes ^2^	[83]
SD-adapted female hamster		• No effects on BW, food intake, circulating insulin/leptin, and hypothalamic metabolic genes	
Male hamster	Single ICV (0.5 or 1.5 µg)	• No effect on food intake regardless of LD or SD	[84]
Female hamster		• Increased food intake in both LD (0.5 µg) and SD (1.5 µg), and NPY expression at 3 h	
Adult male rat	Single ICV (100 or 500 ng)	• Increased food intake at 2 h • No effect on BW at 24 h	[22]
Adult male rat	ICV (1 µg/h) for 5 days	• Increased food and water intake • No effects on whole-body energy expenditure and BAT thermogenesis	[79]
Adult male rat	Single ICV (50 or 250 pmol)	• No effect on food intake	[85]
Adult male rat	Intra-amygdaloid microinjection of RFRP-1 (37.8 pmol)	• Decreased liquid food intake over 1 h • No effect on locomotor activity	[86]
	RF9 (41.4 pmol) + RFRP-1	• Prevents RFRP-1 effect on food intake	
Ovariectomized adult female sheep	ICV (40 µg/h) for 4 h	• Increased food intake at 2, 4 h • No effects on thermogenesis of muscle and visceral WAT	[79]
Adult male cynomolgus macaque monkey	ICV (3 µg/kg/h) for 9 days	• Increased food intake	[79]

^1^ Measured during a short time-period in the dark phase. ^2^ NPY, POMC, and somatostatin expression in the hypothalamus. BW, body weight; BAT, brown adipose tissue; WAT, white adipose tissue; SD or LD, short-day or long-day photoperiod.

**Table 2:** Expression of GnIH or GnIH-Rs in peripheral metabolic tissue.

Tissue	Subject	Expression of GnIH or GnIH-Rs		Method	Ref.
Adipose tissue	Human	Tissue	GPR147/GPR74	RT-PCR	[98]
		Mature adipocyte	GPR147/GPR74	Western	
	Rat	Tissue	GPR147	RT-PCR, Western	[99]
	Mouse	Tissue	GPR147/GPR74	RT-PCR	Figure 1
	Japanese quail	Tissue	GPR147	RT-PCR	Figure 1
	Mouse 3T3-L1 ^1^	Mature adipocyte ^2^	GPR147/GPR74	RT-PCR	[100]
Pancreas	Piglet	Islet	GnIH	Immunostaining	[29]
	Rat	Tissue	GnIH and GPR147	RT-PCR, Western, Immunostaining	[99]
	Mouse	Islet	GPR147	Immunostaining	[101]
	Mouse αTC1 ^1^	α-cells of islet	GPR147	RT-PCR, Immunostaining	[101]
GI tract	Piglet	Esophagus, stomach, small and large intestine	GnIH	Immunostaining	[29]
	Female pig	Intestine	GPR147	RT-PCR	[102]
	Mouse	Stomach, ileum, and colon	GnIH and GPR147	RT-PCR	[103]
Liver	Rat	Tissue	GPR147	RT-PCR, Western	[99]
Skeletal muscle	Rat	Tissue	GPR147	RT-PCR, Western	[99]

^1^ In vitro cell models. ^2^ In vitro-differentiated 3T3-L1 cells.

**Table 3:** Effect of peripheral GnIH administration on metabolism. Chicken GnIH peptide or mammalian RFRP-3 peptide is used for IP injection.

Animal Models	IP Injection of GnIH	Metabolic Effects	Ref.
Female domestic chicken	30 nmol × twice/day for 14 days	• Disrupts the physical and chemical barriers of the intestine • Increased intestinal inflammation	[107]
Male mouse	20 ng, 200 ng, or 2 µg/day for 8 days	• Increased food intake • Increased BW • Increased WAT mass	[104]
Male mouse	20 µg × twice/day for 21 days	• Increased food intake and BW • Increased liver and eWAT mass • Decreased testis mass • Glucose intolerance and insulin resistance	[105]
Rat (male and female mixed population)	1 or 10 µg × twice/day for 14 days	• Increased food intake during photophase • Increased meal frequency • Increased BW • Glucose intolerance and insulin resistance • Increased inflammation in liver, skeletal muscle, or WAT	[99]
Female piglet	0.1 or 1 mg × twice/day for 14 days	• Increased food intake and BW • Increased organ mass in pancreas, pgWAT, iWAT and liver • Glucose intolerance • Altered gene expression in liver, pgWAT, and iWAT related to lipid and glucose metabolism	[106]

eWAT, epididymal white adipose tissue; pgWAT, perigonadal white adipose tissue; iWAT, inguinal white adipose tissue.

## Data Availability

No new data were created or analyzed in this study.
